# Developing neural network diagnostic models and potential drugs based on novel identified immune-related biomarkers for celiac disease

**DOI:** 10.1186/s40246-023-00526-z

**Published:** 2023-08-17

**Authors:** Tao Shen, Haiyang Wang, Rongkang Hu, Yanni Lv

**Affiliations:** Anhui Provincial Key Laboratory of Molecular Enzymology and Mechanism of Major Diseases, Key Laboratory of Biomedicine in Gene Diseases, Health of Anhui Higher Education Institutes, College of Life Sciences, Anhui Normal University, Wuhu, China

**Keywords:** Celiac disease, Immune-related genes, Machine learning algorithms, Artificial neural network, Molecular docking method, Potential targeted drugs, Immune genes score

## Abstract

**Background:**

As one of the most common intestinal inflammatory diseases, celiac disease (CD) is typically characterized by an autoimmune disorder resulting from ingesting gluten proteins. Although the incidence and prevalence of CD have increased over time, the diagnostic methods and treatment options are still limited. Therefore, it is urgent to investigate the potential biomarkers and targeted drugs for CD.

**Methods:**

Gene expression data was downloaded from GEO datasets. Differential gene expression analysis was performed to identify the dysregulated immune-related genes. Multiple machine algorithms, including randomForest, SVM-RFE, and LASSO, were used to select the hub immune-related genes (HIGs). The immune-related genes score (IG score) and artificial neural network (ANN) were constructed based on HIGs. Potential drugs targeting HIGs were identified by using the Enrichr platform and molecular docking method.

**Results:**

We identified the dysregulated immune-related genes at a genome-wide level and demonstrated their roles in CD-related immune pathways. The hub genes (*MR1*, *CCL25*, and *TNFSF13B*) were further screened by integrating several machine algorithms. Meanwhile, the CD patients were divided into distinct subtypes with either high- or low-immunoactivity using single-sample gene set enrichment analysis (ssGSEA) and consensus clustering. By constructing IG score based on HIGs, we found that patients with high IG score were mainly attributed to high-immunoactivity subgroups, which suggested a strong link between HIGs and immunoactivity of CD patients. In addition, the novel constructed ANN model showed the sound diagnostic ability of HIGs. Mechanistically, we validated that the HIGs play pivotal roles in regulating CD's immune and inflammatory state. Through targeting the HIGs, we also found potential drugs for anti-CD treatment by using the Enrichr platform and molecular docking method.

**Conclusions:**

This study unveils the HIGs and elucidates the networks regulated by these genes in the context of CD. It underscores the pivotal significance of HIGs in accurately predicting the presence or absence of CD in patients. Consequently, this research offers promising prospects for the development of diagnostic biomarkers and therapeutic targets for CD.

**Supplementary Information:**

The online version contains supplementary material available at 10.1186/s40246-023-00526-z.

## Background

Celiac disease (CD) is the most common autoimmune enteropathy worldwide that mainly occurs in genetically susceptible individuals who develop an immune response to gluten [1]. Gluten is found in almost all cereals, such as wheat, barley, and rye, making it the leading environmental factor in its pathogenesis [2]. CD patients have wide-ranging clinical manifestations and onsets, including classical intestinal-related symptoms (diarrhoea, failure to thrive) and non-intestinal manifestations (anaemia, dermatitis, osteoporosis), that often lead to a delay in CD diagnosis [3]. Currently, the only clinical treatment for CD is strict adherence to a gluten-free diet (GFD), which effectively relieves symptoms of intestinal inflammation and promotes intestinal microvilli regrowth. However, on the one hand, a GFD is very difficult to achieve given the ubiquity of gluten as a common food additive, as well as due to dietary habits, high costs of the GFD and the social restrictions it imposed on patients [4]. On the other hand, a GFD can be associated with several disadvantages, mainly including psychological problems in patients, decreased quality of life, fear of mandatory GFD [5], metabolic syndrome, possible vitamin and mineral deficiencies, increased cardiovascular risk, and constipation [6]. Thus, it is necessary to explore the molecular characteristics and mechanism of CD development, which can provide new strategies for diagnosing and treating this disease.

In celiac disease, dietary gluten triggers a T cell-driven small intestinal inflammation in a subset of genetically predisposed subjects carrying the human leukocyte antigen (HLA) DQ2 and/or DQ8 haplotype [2]. HLA DQ2/DQ8 can bind gluten peptides and trigger host responses such as innate and adaptive immune responses and increased intestinal permeability [7]. However, the presence of a specific HLA accounted only for about 40% of the genetic predisposition, indicating that these genes are necessary but insufficient for CD to develop and leaving most of the genes involved in the development of CD still unknown [8]. Although the aetiology and pathophysiology of CD are not fully understood, the condition is caused by a combination of environmental, genetic, and immunological factors [9]. In this direction, whole genome-wide revealing the dysregulated immune-related genes are hopeful to identify distinct gene expression signatures that could help to stratify patients with CD, or highlight new pathways implicated in CD development.

To systematically identify hub immune-related genes involved in CD, we integrated multiple machine learning algorithms and identified *MR1*, *CCL25*, and *TNFSF13B* as the hub immune-related genes (HIGs). Based on these HIGs, we constructed an immune genes score (IG score) to assess the risk of CD. Meanwhile, we found that most high IGscore patients were also characterized by high immunoactivity. Further gene set enrichment analysis (GSEA) showed that the HIGs are dramatically enriched in immune-related pathways, including the intestinal immune network for IgA production, a significant driving force for CD development. Based on these HIGs, we also constructed a novel ANN model, which showed good accuracy for CD diagnosis in training and test cohorts. In addition, we also revealed the potential drugs that target HIGs. Together, these results will expand our understanding of the functional characteristics of immune-related genes involved in CD progression and provide potential diagnostic biomarkers and therapeutic targets.

## Results

### Identification of differentially expressed immune-related genes in CD

**Fig. 1:**
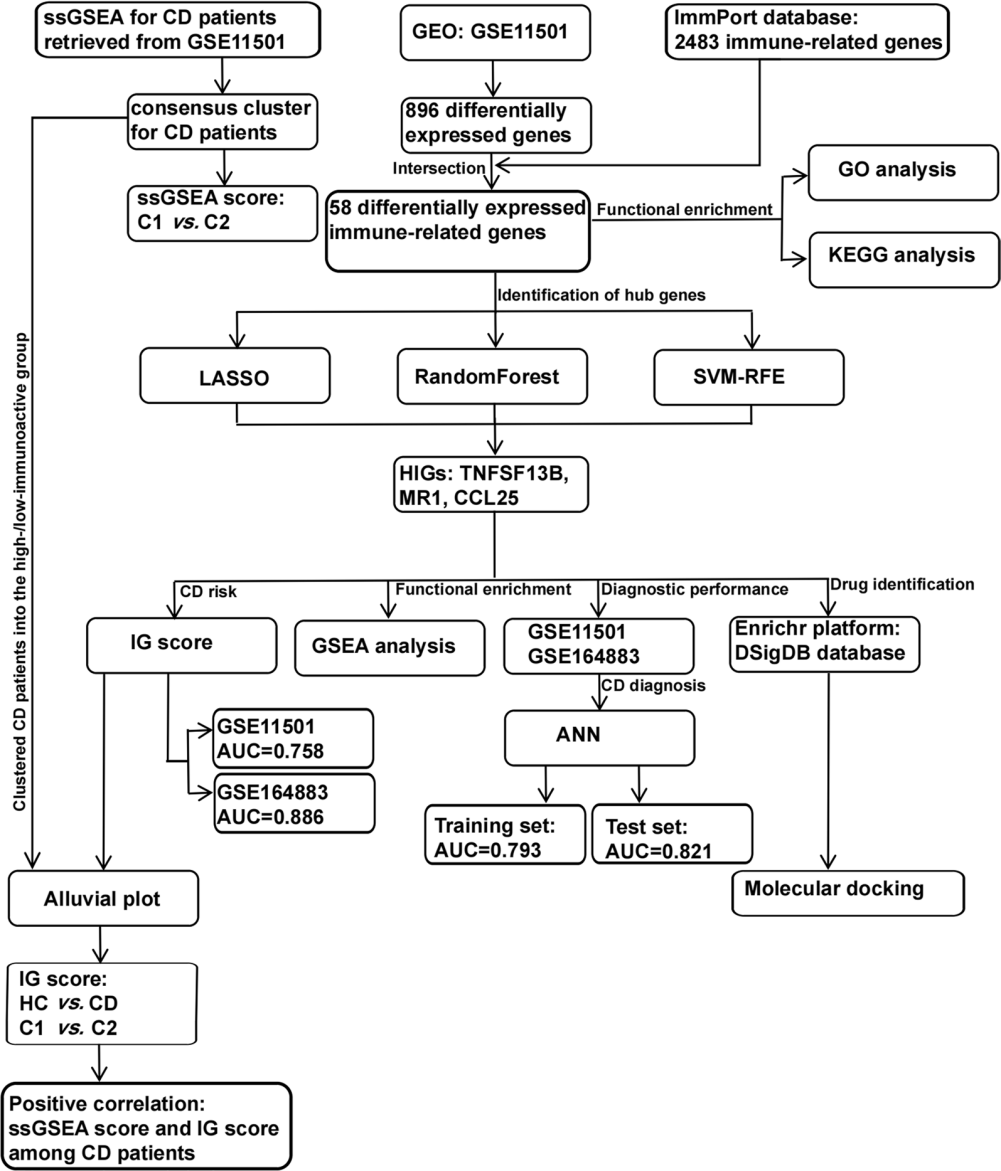
Schematic view of the procedures for data collection and analyses in celiac disease. HC represents healthy control; CD represents celiac disease; C1 and C2 are stratified by the ssGSEA score of celiac disease patients. C1 represents high-immunoactivity patients, while C2 represents low-immunoactivity patients. HIGs represents hub immune-related genes

**Fig. 2:**
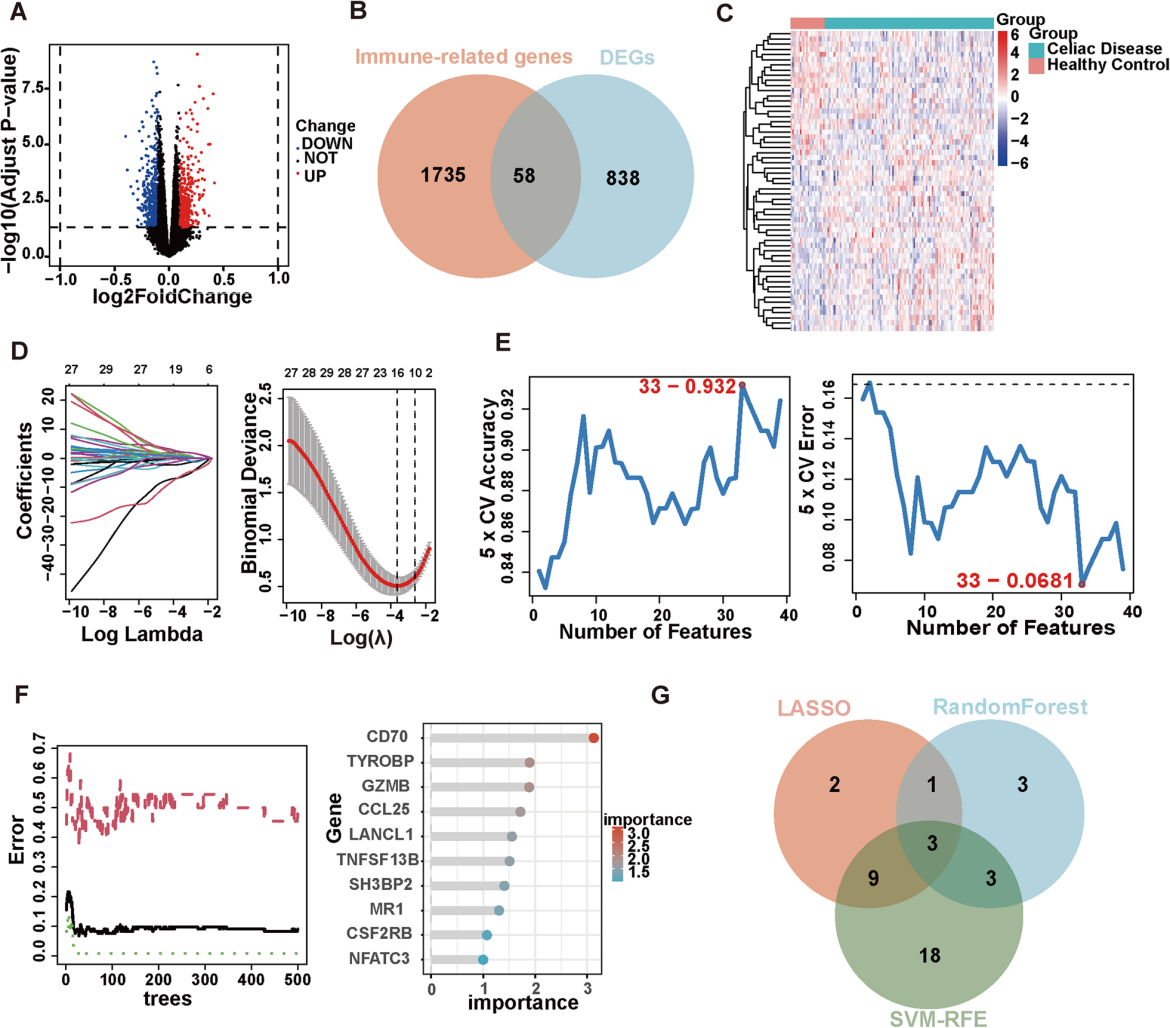
Identification of the hub immune genes (HIGs). **A** The volcano plot shows the differentially expressed genes (DEGs) in celiac disease retrieved from GSE11501. **B** Venn plot shows the intersection of DEGs with immune-related genes. **C** Heatmap shows the overall landscape of 58 differentially expressed immune-related genes between healthy control and celiac disease.** D** LASSO coefficient profiles of the indicated differentially expressed immune-related genes (left panel). After cross-validation for tuning parameter selection, 15 candidate HIGs were identified (right panel). **E** SVM–RFE algorithm identified 33 candidate HIGs with an accuracy of 0.932 (left panel) and an error of 0.0681 (right panel). **F** RandomForest algorithm identified 10 candidate HIGs. RandomForest error rate versus the number of classification trees (left panel) and gene importance scores (right panel). **G** Venn plot shows the overlapped candidate HIGs

We conducted a set of analyses to investigate the role of immune-related genes in celiac disease systematically. The study design is illustrated in Fig. 1. We downloaded the RNA-seq datasets from 110 CD patients and 22 healthy controls retrieved by GEO datasets (GSE11501) and performed differential gene expression analysis. Our results identified 896 differentially expressed genes, of which 369 are up-regulated and 527 are down-regulated (Fig. 2A) (Additional file 1: Table S1). To further screen the immune-related genes among the indicated differentially expressed genes, we intersected the 896 differentially expressed genes with 2483 immune-related genes annotated by the ImmPort database (https://www.immport.org/resources), and finally obtained 58 differentially expressed immune-related genes (Fig. 2B). The expression profiles of the differentially expressed immune-related genes are shown in Fig. 2C. To investigate the role of these genes, we performed Gene Ontology (GO) and Kyoto Encyclopedia of Genes and Genomes (KEGG) analysis. Consequently, GO enrichment analysis revealed multiple immune-related biological processes, including “T cell and B cell activation”, “T-helper cell differentiation”, “interleukin-6/-8 production”, “interleukin-15-mediated signalling pathway”, “MHC class II protein complex binding”, “antigen processing and presentation of exogenous peptide antigen via MHC class II”, “MyD88-dependent toll-like receptor signalling pathway”, “G protein-coupled receptor binding”, “peptidyl-tyrosine autophosphorylation”, “regulation of cytokine production involved in inflammatory response”, “regulation of acute inflammatory response to antigenic stimulus”, “chemokine (C-X-C motif) ligand 2 production”, and “regulation of NLRP3 inflammasome complex assembly” (Additional file 2: Fig. S1A). The KEGG enrichment analysis again enriched the indicated differentially expressed immune-related genes in multiple immune-related pathways, including “T cell receptor signalling pathway”, “B cell receptor signalling pathway”, “Th1 and Th2 cell differentiation”, “Th17 cell differentiation”, “Natural killer cell-mediated cytotoxicity”, “EGFR tyrosine kinase inhibitor resistance”, “Intestinal immune network for IgA production”, and “PD-L1 expression and PD-1 checkpoint pathway in cancer” (Additional file 2: Fig. S1B).

### Identification of hub immune-related genes (HIGs)

Next, to find out the hub differentially expressed immune-related genes in CD, we applied 3 machine learning algorithms, including least absolute shrinkage and selection operator (LASSO), support vector machine recursive feature elimination (SVM-RFE), and random forest. Firstly, we utilized the LASSO algorithm to identify the variation in regression coefficients of 58 differentially expressed immune-related genes (Fig. 2D), and eventually, 15 candidate genes were screened. We also established the SVM-RFE model to screen out the genes with the minimum cross-validation error (Fig. 2E), and the SVM-RFE algorithm eventually screened 33 candidate genes with an accuracy of 0.932 and an error of 0.0681. Besides, the differentially expressed immune-related genes were also incorporated into the random forest model, and the cross-validation error was minimized to 28 trees (Fig. 2F). Subsequently, 10 candidate genes with important points larger than one was eventually identified by random forest (Additional file 1: Table S2). In summary, the LASSO algorithm identified 15 candidates, the SVM-RFE algorithm identified 33 candidates, and the randomForest algorithm identified 10 candidates (Table 1). By intersecting all the candidates (Fig. 2G), we found that *MR1*, *CCL25*, and *TNFSF13B* could be identified by the three machine learning approaches and thus defined as hub immune-related genes (HIGs).

**Table 1:** Scanning of candidate machines by 3 machine learning algorithms

Methods	Genes
Lasso	*CTSS, MR1, PSMC1, PSMC4, PSMD11, SLPI, ORM1, CCL25,* *UNC93B1, LTBP2, SCGB3A1, TNFSF13B, ANGPTL3, TYROBP, MIF*
RandomForest	*TYROBP, GZMB, CCL25, LANCL1, TNFSF13B, SH3BP2, MR1,* *CSF2RB, NFATC3, CD70*
SVM-REF	*MIF, CCL25, LCP2, ANGPTL3, MR1, ORM1, SCGB3A1, GZMB,* *PDK1, PSMC4, SLPI, UNC93B1, TMSB10, HTR3B, LCK, CBL,* *PPP4C, CKLF, ISG20, CSF2RB, IRF7, AP3B1, CRLF3, CTSS,* *CD70, TNFSF13B, ULBP3, MAPK3, CKLF, NR1D2, TNFSF14,* *RARA, PSMC1, EIF2AK2*

### CD patients were stratified into subgroups with distinct immunoactivities

**Fig. 3:**
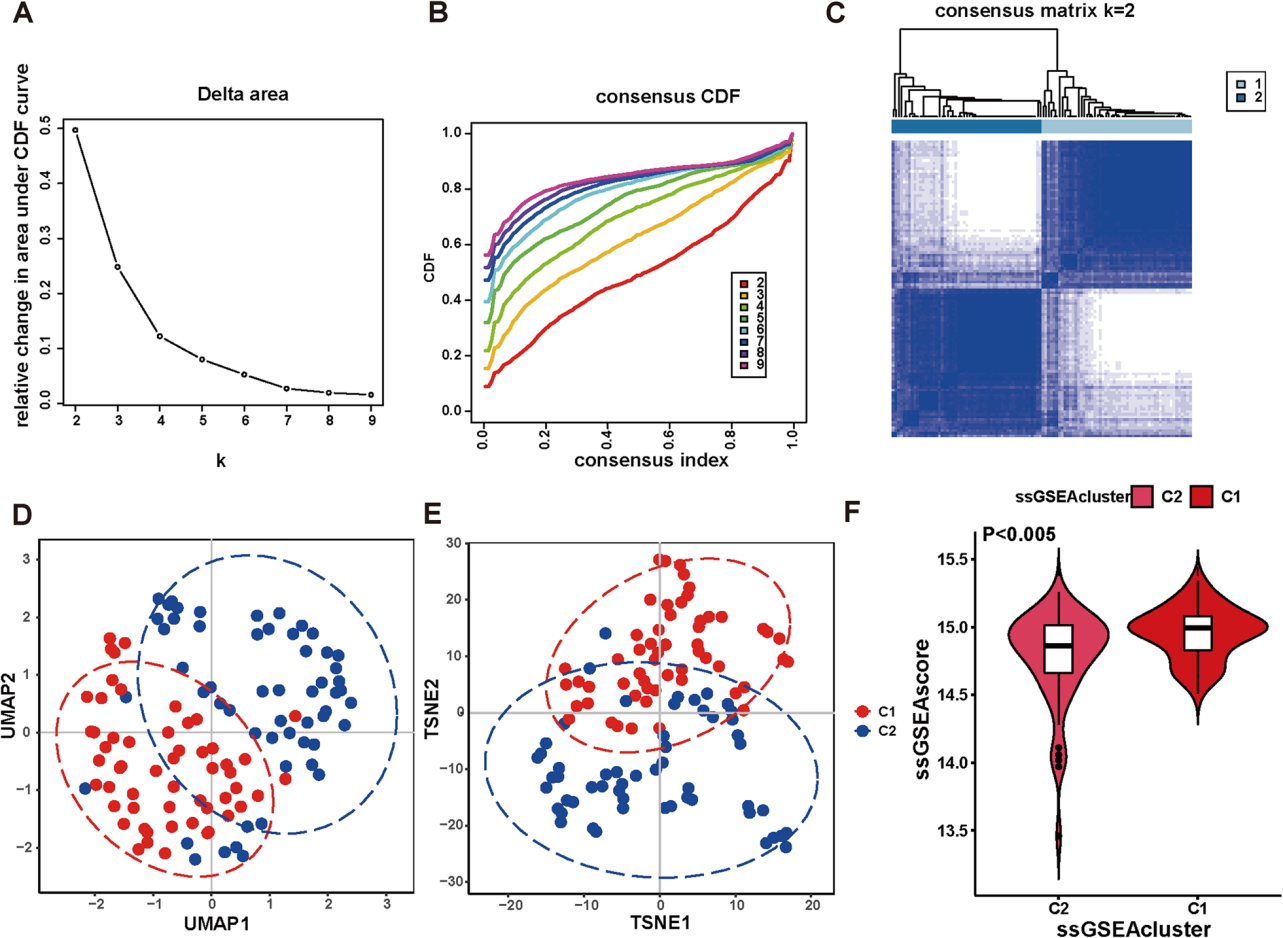
Consensus clustering based on the ssGSEA score to stratify distinct immunoactivity patients. **A, B** relative change in area under cumulative distribution function (CDF) curve for *k *= 2 to 9 (**A**). Consensus clustering CDF for *k *= 2 to 9 (**B**). **C** Consensus clustering heatmap when *K *= 2. Related to Additional file 2: Fig. S2. **D, E** Each dot represents a single sample of UMAP (**D**) and t-SNE (**E**) analysis for the GSE11501-retrieved celiac disease patients. **F** The violin plot shows the ssGSEA score of the indicated GSE11501-retrieved celiac disease patients distributed in the C1 and C2 groups

Celiac disease is an autoimmune disease in which the immune system plays a central role in its pathogenesis, suggesting that CD patients' immunoactivity is closely related to CD development. Thus, to assess the relationship between CD patients' immunoactivity and CD risk, we performed ssGSEA and consensus clustering. We performed the ssGSEA of 28 immune gene sets annotated by the TISIDB database (http://cis.hku.hk/TISIDB/). The ssGSEA score of 28 immune gene sets in each CD patient was calculated (Additional file 1: Table S3, Additional file 2: Fig. S2). We classified GSE11501-retrieved CD patients using the k-means of unsupervised consensus clustering based on the ssGSEA score of 28 immune gene sets. The optimal cluster number was then determined as *K *= 2 (Fig. 3A–C and Additional file 2: Fig. S3). As shown in Fig. 3A–C, in the case of *k *= 2, CD patients were divided into two subgroups, C1 and C2, with clear boundaries, suggesting a stable and reliable clustering. Following that, UMAP and t-SNE analyses were performed to validate the subtype assignments, and results from both methods indicated that samples in one subgroup were more similar to each other than samples in the other (Figs. 3D, E).

Upon comparing the ssGSEA score of 28 immune gene sets between the C2 and C1 subgroups, we observed significant differences in the landscape of 15 immune cells. Notably, the C1 subgroup exhibited notably higher ssGSEA score for immune cells such as "Central memory CD8 T cell," "Natural killer cell," "Natural killer T cell," "Activated dendritic cell," "Plasmacytoid dendritic cell," "Macrophage," "Eosinophil," "Mast cell," and "Neutrophil" (Additional file 2: Fig. S4). Furthermore, we examined the relationship between the immunoactivity of the C1 and C2 subgroups by comparing their respective ssGSEA score. Our findings demonstrated a substantially higher ssGSEA score for the C1 subgroup compared to the C2 subgroup (Fig. 3F), indicating that the overall immunoactivity of the C1 subgroup surpassed that of the C2 subgroup. Consequently, based on their immune characteristics, we were able to classify CD patients into two distinct subgroups: the high-immunoactivity group and the low-immunoactivity group.

### Construction and validation of the IG score based on HIGs

**Fig. 4:**
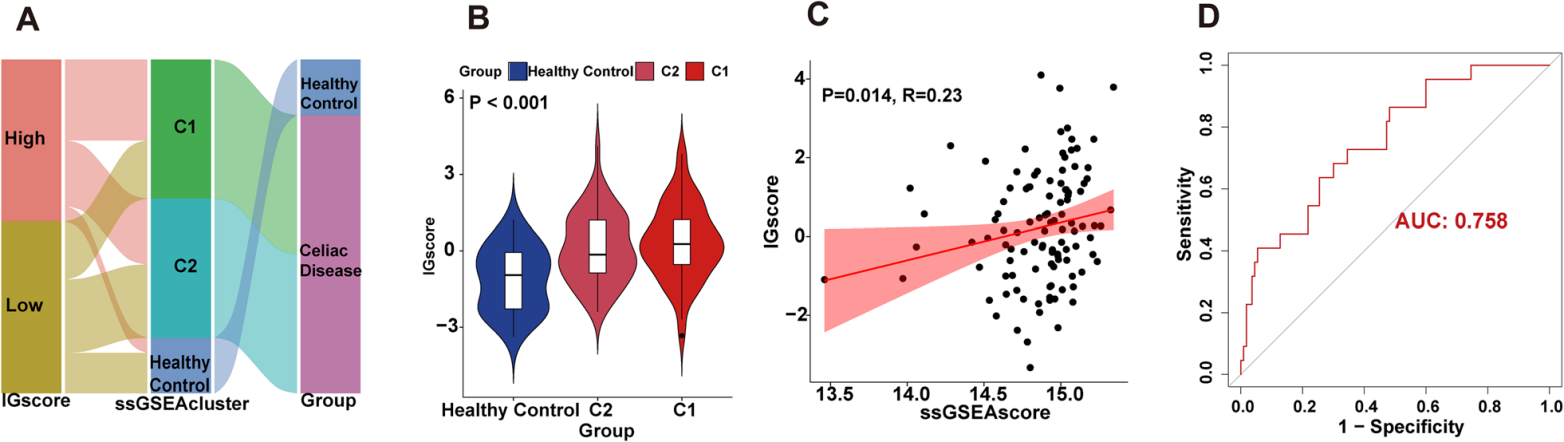
Construction and evaluation of the IG score on HIGs. **A** The alluvial plot shows the connection between IG score groups, ssGSEA score groups, healthy control, celiac disease and healthy control. **B** The violin plot shows the IG score of healthy control and patients distributed in the C1 and C2 groups. **C** Correlation analysis between IG score and ssGSEA score. **D** The ROC curve of the IG score

Based on the HIGs mentioned above (*MR1*, *CCL25*, and *TNFSF13B*), we calculated the IG score, which was further used to assess the risk of developing CD for each sample retrieved from GSE11501 using principal component analysis. All samples retrieved from GSE11501 were classified as low-IG or high-IG score subgroups according to the IG score < or > 0 (Additional file 1: Table S4). Meanwhile, we proceeded to a correlation analysis to assess the relevance of IG score and immunoactivity. By visualizing the basic profiles of each sample in alluvial plots, which included high- and low-IG score groups and high- and low-immunoactivity patients divided by ssGSEA mentioned above analyses. Our results showed that most CD patients with high IG scores belong to the C1 subgroup, while most CD patients with low IG score belong to the C2 subgroup (Fig. 4A). Subsequently, we also compared the IG score among the three subgroups, including healthy control, the C1 subgroup, and the C2 subgroup. The results showed that the IG score of patients in the C1 subgroup was higher than in the C2 subgroup, and the IG score of patients with CD (C1 and C2) was higher than healthy control (Fig. 4B). In addition, when we performed correlation analysis, we found a positive correlation between the IG score and the ssGSEA score (Fig. 4C). We executed a ROC analysis to further evaluate the IG score's predictable power. As the area under the IG score's ROC curve (AUC) was 0.758, the result indicated the IG score has a well-predictable performance (Fig. 4D). Together, these data not only indicated a predictable power of the IG score based on HIGs to identify the risk of individuals developing CD, but also built a strong connection between HIGs and immunoactivities of CD patients.

In addition, we evaluated whether the IG score based on HIGs has a similar predictable value in another independent CD patient cohort. We included CD patients from GSE164883 in the IG score analyses (Additional file 1: Table S5). Similarly, we found that the IG score of CD patients was higher than the control (Additional file 2: Fig. S5A). The AUC score of 0.886 also showed a predictable performance (Additional file 2: Fig. S5B). These results again suggested that the IG score based on HIGs has well sensitivity and predictability.

### HIGs play pivotal roles in contributing to the inflammatory state of CD

**Fig. 5:**
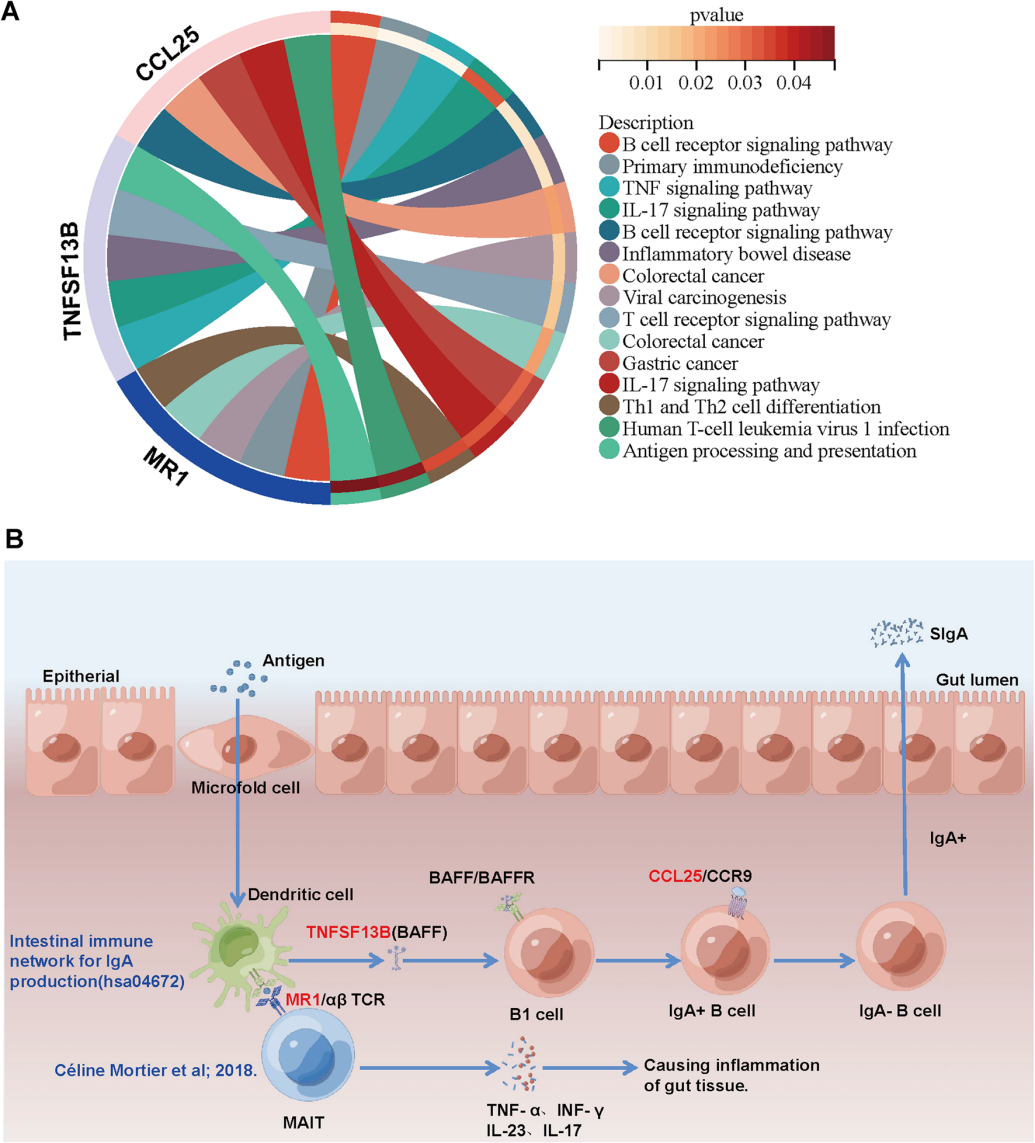
Functional enrichment of the indicated HIGs. **A** GSEA analysis of *MR1*, *TNFSF13B*, and *CCL25*. **B** Schematic illustration of HIGs promoting intestinal inflammation. *CCL25* and *TNFSF13B* were enriched in hsa04672 -Intestinal immune network for IgA production. Besides, Céline Mortier et al. found that *MR1* promotes intestinal inflammation by activating MAIT to produce pro-inflammatory factors

To further investigate the regulatory roles of HIGs, we conducted GSEA analysis to identify the HIGs-regulated signalling pathways. The results showed that *CCL25* was significantly enriched in “B cell receptor signalling pathway”, “Colorectal cancer”, “Gastric cancer”, “IL-17 signalling pathway” and “Human T-cell leukaemia virus 1 infection”. *TNFSF13B* was significantly associated with “TNF signalling pathway”, “IL-17 signalling pathway”, “Inflammatory bowel disease”, “T cell receptor signalling pathway”, and “Antigen processing and presentation”. *MR1* has significantly associated with “B cell receptor signalling pathway”, “Primary immunodeficiency”, “Viral carcinogenesis”, “Colorectal cancer”, and “Th1 and Th2 cell differentiation” (Fig. 5A). In conclusion, we found that HIGs can regulate immune cell receptor signalling pathways, such as the B cell or T cell receptor signalling pathways. Among them, *CCL25* and *TNFSF13B* can regulate IL-17 signalling pathways, notably IL-17 as pro-inflammatory cytokines which promote the chronic inflammatory state characteristic during CD development [10]. *TNFSF13B* can regulate the TNF signalling pathway and possibly trigger inflammatory bowel disease. *MR1* can regulate Th1 and Th2 cell differentiation, which produces abundant pro-inflammatory cytokines.

To reveal the regulator roles of HIGs, we further integrated the KEGG pathway analysis and literature reports and drew the major involvement of HIGs in CD development in the Figdraw platform (Fig. 5B). We found that *CCL25* and *TNFSF13B* were enriched in hsa04672 -Intestinal immune network for IgA production. The activated T helper cells in the pathogenesis of CD will activate B cells to induce them to produce IgA antibodies against tissue transglutaminase, gliadin and endomysium, which exacerbate the chronic inflammatory state characteristic of CD. It has also been shown that *MR1* is expressed on dendritic cells (DCs), which present antigens to mucosal-associated invariant T (MAIT) cells, leading to MAIT activation and production of pro-inflammatory cytokines, such as tumour necrosis factor (TNF)-α, interferon (INF)-γ, interleukin (IL)-23, IL-17 [11]. Subsequently, these mediators will recruit and activate other immune cells, contributing to the induction of gut tissue inflammation.

### Construction and validation of ANN model

To verify the diagnostic roles of the HIGs, we detected the receiver operating characteristic (ROC) curve of each HIG in diagnostic test assessment for both the GSE11501 training set and the GSE164883 validation set (Additional file 2: Fig. S6). With the AUC score of *MR1* = 0.696, *CCL25* = 0.860, and *TNFSF13B* = 0.839, we found that all these HIGs could discriminate CD from healthy controls with higher accuracy in the GSE11501 training set (Additional file 2: Fig. S6A). Following the GSE11501 training set, the AUC score was also calculated in an independent GSE164883 validation set. With the AUC score of *MR1* = 0.741, *CCL25* = 0.906, *TNFSF13B* = 0.988, we also found that all these HIGs could discriminate CD from healthy controls with higher accuracy in the GSE164883 validation set (Additional file 2: Fig. S6B). Besides, we also constructed ANN based on HIGs to diagnose the onset of CD.

**Fig. 6:**
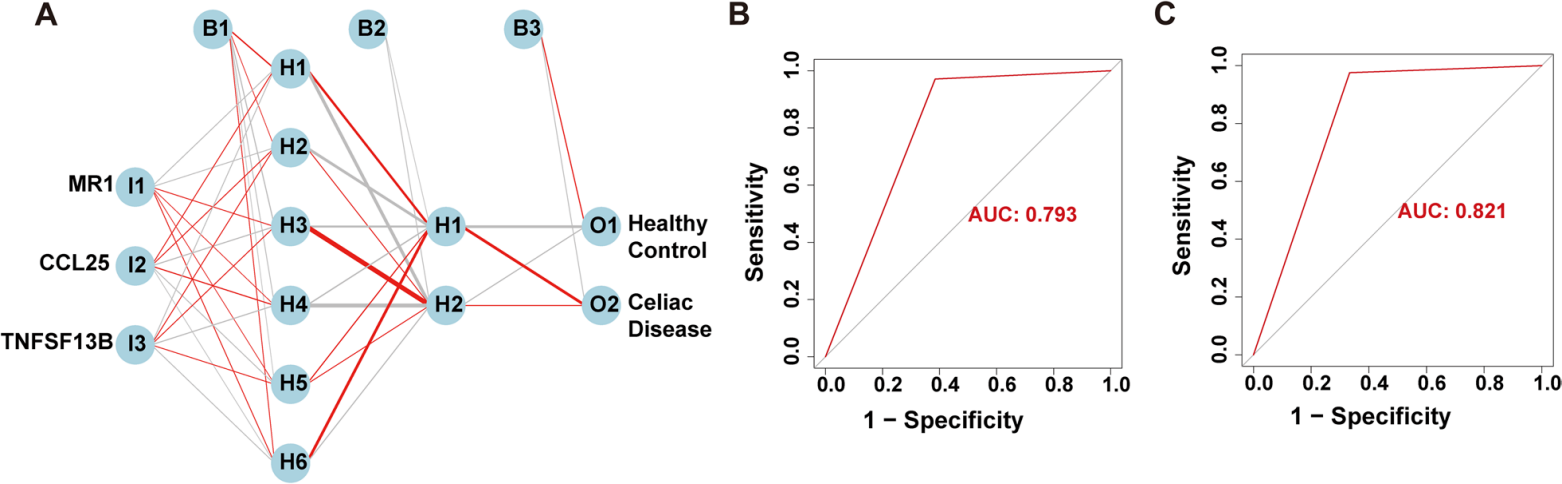
Construction of ANN based on HIGs.** A** Construction of ANN based on *MR1*, *TNFSF13B*, and *CCL25*. **B** The AUC of the training cohort with a value of 0.793. **C** The AUC of the test cohort with a value of 0.821

ANN stands out as a prominent form of artificial intelligence extensively utilized across various specialized domains within clinical medicine. Notably, numerous studies have underscored the remarkable potential of ANN in diagnosing and treating gastrointestinal diseases [12–14]. In line with these findings, we integrated HIGs into an ANN framework to develop a predictive model capable of discerning whether samples belonged to the healthy control or CD groups. The ANN model encompassed three fundamental components: the input layer, hidden layer, and output layer (Fig. 6A). Subsequently, we compared the predictions generated by the ANN model with the actual grouping information of the samples. The accuracy of the ANN predictions for the training and test sets is presented in Table 2, yielding values of 0.9146 and 0.92, respectively. Moreover, we employed Receiver Operating Characteristic (ROC) analysis to evaluate the predictive capability of the ANN model on both the training and test sets. The area under the curve (AUC) value for the training set was 0.793 (Fig. 6B), while the AUC value for the test set was 0.821 (Fig. 6C). Additionally, we constructed an ANN model (Additional file 2: Fig. S7A) to diagnose the C1 and C2 subsets, aiming to identify CD patients with varying degrees of risk severity. Additional file 1: Table S6 showcases the accuracy of the ANN predictions for the training and test sets, which yielded values of 0.8171 and 0.7143, respectively. The ROC analysis further demonstrated the prediction capability of the ANN model, with an AUC value of 0.824 for the training set (Additional file 2: Fig. S7B) and an AUC value of 0.733 for the test set (Additional file 2: Fig. S7C). In summary, the ANN model exhibits substantial promise and can potentially serve as an independent diagnostic predictor for CD.

**Table 2:** ANN diagnosis effect for the training and test sets

	Training set		Test set	
	Healthy control	Celiac disease	Healthy control	Celiac disease
*Prediction*				
Healthy control	8	5	6	3
Celiac disease	2	67	1	40
Accuracy	0.9146		0.92	
AUC	0.793		0.821	

### Identification and docking of potential drugs targeting HIGs

**Fig. 7:**
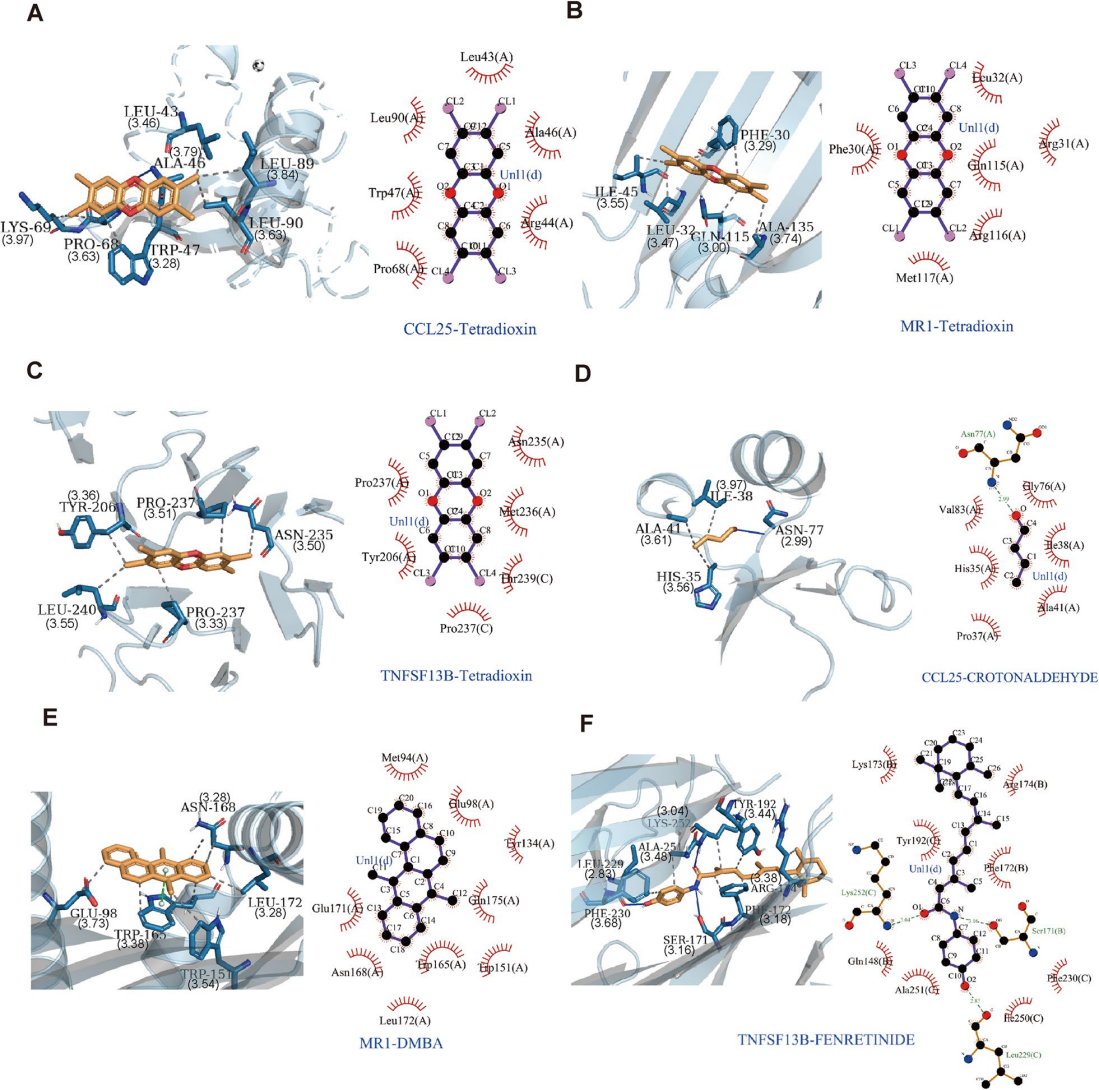
3D (left) and 2D (right) views of the interacted interface between HIGs and their top two potential binding drugs. **A–C** The structure of the complex formed by the docking of Tetradioxin with CCL25, MR1 and TNFSF13B.** D** The structure of the complexes formed by the docking of CROTONALDEHYDE with CCL25. **E** The structure of the complex formed by the docking of DMBA with MR1. **F** The structure of the complex formed by the docking of FENRETINIDE with TNFSF13B. The indicated drugs were selected according to the absolute values of the binding energy to CCL25, MR1 and TNFSF13B

To find out the drugs targeting HIGs, we used the Enrichr platform (https://maayanlab.cloud/Enrichr/) for online analysis and screening. We identified seven drugs targeting HIGs based on the DSigDB database with a *p* value < 0.05 (Table 3). Next, we used the molecular docking method (MDM) to investigate the binding affinity of the drugs with their targeting HIGs, and their binding energy is shown in Table 4. The results showed that Tetradioxin was able to target all the HIGs, and the absolute values of the binding energy of the three HIGs proteins to Tetradioxin were higher than those to the other drug molecules (Fig. 7A–C), in the order of *CCL25*-Tetradioxin (− 5.6 kcal/mol), *MR1*-Tetradioxin (− 6.8 kcal/mol), and *TNFSF13B*-Tetradioxin (− 6.62 kcal/mol). Among them, the amino acid binding sites LEU-43 and TRP-47 of *CCL25* were at the nearest distances of 3.46 Å and 3.28 Å, respectively, from Tetradioxin, while PRO-68, LYS-69, LEU-89 and LEU-90 were relatively distant from Tetradioxin. The amino acid binding sites TYR-206, ASN-235 and LEU-240 of the *TNFSF13B* were all distant from Tetradioxin, and only PRO-237 was nearer to Tetradioxin with a distance of 3.33 Å. In contrast, among the seven amino acid binding sites of *MR1*, PHE-30 had three hydrogen bond-forming interactions with Tetradioxin, LEU-32 was at a distance of 3.47 Å from Tetradioxin, ILE-45 had a distance of 3.55 Å with Tetradioxin, GLN-115 had a distance of 3.00 Å with Tetradioxin, and ALA-135 had a distance of 3.74 Å with Tetradioxin. Overall, the distance of the nearest amino acid binding site (GLN-115 3.00 Å) between *MR1* and Tetradioxin was shorter than the nearest distances of *CCL25* (TRP-47 3.28 Å) and *TNFSF13B* (PRO-237 3.33 Å). Thus, in terms of the distance of the HIGs amino acid binding sites from Tetradioxin, there are multiple and nearer amino acid binding sites between *MR1* and Tetradioxin, and these amino acids can form more interactions, that is, *MR1* exhibits a stronger binding energy of 6.8 (kcal/mol) relative to *CCL25* and *TNFSF13B*. In addition, the absolute values of the binding energies of the three complexes, *CCL25*-CROTONALDEHYDE (− 3.03 kcal/mol), *MR1*-DMBA (− 6.8 kcal/mol) and *TNFSF13B*-FENRETINIDE (− 6.56 kcal/mol), were relatively high (Fig. 7D–F), while four complexes, *MR1*-Demecolcine (− 5.94 kcal/mol) and *MR1*-cyclophosphamide (− 4.64 kcal/mol), and *TNFSF13B*- Demecolcine (− 6.01 kcal/mol) and *TNFSF13B*- diuron (− 5.4 kcal/mol), four complexes with relatively low absolute values of binding energy (Additional file 2: Fig. S8), suggesting that these drugs may have a regulatory effect on HIGs.

**Table 3:** Potential drugs targeting HIGs

Drugs	*P* value	Target genes (HIGs)
Tetradioxin CTD 00006848	0.006682774	*CCL25; MR1;TNFSF13B*
DMBA CTD 00007046	0.011059423	*MR1*
diuron CTD 00005864	0.0212979	*TNFSF13B*
Demecolcine CTD 00005762	0.005581458	*MR1; TNFSF13B*
CROTONALDEHYDE CTD 00000669	0.027348037	*CCL25*
FENRETINIDE CTD 00007166	0.033813077	*TNFSF13B*
Cyclophosphamide CTD 00005734	0.041416571	*MR1*

**Table 4:** The estimated binding energy (kcal/mol) of HIGs-drugs

Drugs	*CCL25*	*MR1*	*TNFSF13B*
Tetradioxin CTD 00006848	− 5.6	− 6.8	− 6.62
CROTONALDEHYDE CTD 00000669	− 3.03	–	–
DMBA CTD 00007046	–	− 6.8	–
Demecolcine CTD 00005762	–	− 5.94	− 6.01
Cyclophosphamide CTD 00005734	–	− 4.64	–
FENRETINIDE CTD 00007166	–	–	− 6.56
diuron CTD 00005864	–	–	− 5.4

## Discussion

CD is an autoimmune disease typically characterized by chronic small intestine inflammation [15]. The incidence and prevalence of CD have increased over time, and worse, it can occur at any age, from infancy to older people [3]. However, the diagnosis of CD remains challenging for clinical practice. On the one hand, the digestive tract is beyond our visual observation. On the other hand, CD is a multifactor disease, and the symptoms of CD are currently unclear. A single diagnostic method based on imaging examinations heavily relying on radiologists' experiences is limited. The application of ANN based on gene expression signatures has gradually broken through this dilemma. One strong piece of evidence is that ANN has excellent potential in diagnosing gastrointestinal diseases closely related to CD [12]. For another, this state-of-the-art technique exhibits excellent performance in diagnosis, prognostic prediction, and treatment in many other diseases, and some evaluation indexes of ANN models even achieved an accuracy of 100% [16, 17]. Therefore, we incorporated HIGs into the ANN and constructed an ANN model to predict whether the samples belonged to healthy control or CD in the present study, and the prediction accuracy for the training and test sets are 0.9146 and 0.92, respectively. Meanwhile, we evaluated the prediction capability of the ANN model on the training and test sets using the ROC curves, where the AUC value for the training set is 0.793, and the AUC value for the test set is 0.821. That is, ANN based on the HIGs’ expression levels has the potential to be used as an independent diagnostic predictor for CD.

Differential gene expression analysis first identified the HIGs used for building ANN models. We obtained 58 differentially expressed immune-related genes between healthy controls and CD patients. The enrichment analysis of these genes suggests that B and T's cells play a pivotal role in the pathogenesis of CD. Then, we applied 3 machine learning algorithms, including LASSO, SVM-RFE, and random forest, to identify the hub immune-related genes (HIGs). We found that the machine learning identified *MR1, CCL25,* and *TNFSF13B*, indicating the central role of *MR1*, *CCL25*, and *TNFSF13B* in B and T cell regulation and CD progression. Indeed, our GSEA and KEGG pathway analyses enriched these HIGs in immune-/inflammatory-related functions and pathways.

Meanwhile, literature reports have also shown the critical roles of the HIGs in regulating inflammatory bowel disease's immune and inflammatory state [11, 18, 19]. For example, *CCL25* is a thymus-expressed chemokine expressed mainly in the thymus and epithelial cells of the small intestinal villi lining. The interaction of *CCL25* and its receptor is involved in T cell development and gut-associated immune responses [20, 21], as well as participating in various inflammatory diseases and contributing to inflammatory responses, including inflammatory bowel disease [22]. *MR1* can specifically recognize small metabolite antigens and present antigens to activate T cells [23, 24]. Once *MR1* binds antigens, the *MR1*-antigen complex is revealed on the cell surface and is recognized by mucosal-associated invariant T (MAIT) cells which could produce the pro-inflammatory cytokines. These pro-inflammatory cytokines will recruit and activate other immune cells to contribute to the inflammation of gut tissue [11]. *TNFSF13B* can induce B cell proliferation, differentiation and immunoglobulin production. Once the expression of *TNFSF13B* is dysregulated, it disrupts B cell self-tolerance, leading to autoimmune diseases and B cell malignancies [25–27]. In summary, our GESA and KEGG pathway analyses and the literature reports have all shown that the HIGs play pivotal roles in regulating CD progression.

As an autoimmune disease, the immune characteristics of different models can provide a theoretical basis for classifying the immune subtypes of CD. Therefore, immune subtypes of CD were also identified using “ConsensusClusterPlus” package based on the ssGSEA score of 28 immune gene sets in this paper. According to the ssGSEA score of 28 immune gene sets, the immune subtypes of CD were divided into 2 clusters and defined as C1 and C2. Among them, C1 is the high-immunoactivity group, and C2 is the low-immunoactivity group. The correlation between HIGs and 2 immune subtypes of CD was further analysed. Also, we constructed the IG score based on HIGs to assess the CD risk. The results showed that the CD risk of patients in the C1 subgroup was higher than in the C2 subgroup, and the CD risk of patients with CD (C1 and C2) was higher than healthy controls, which suggested that HIGs could be a predictor for the immunoactivity and the risk of CD.

Considering the critical roles of HIGs in regulating CD's immune and inflammatory state, we start to seek potential drugs for CD treatment by targeting HIGs. Knowing the only clinical treatment for CD is strict adherence to a GFD currently. However, research studies have shown that approximately half of patients with CD are dissatisfied with the GFD and want to seek treatments that can replace the GFD [28]. As mentioned above, the HIGs might function at the key nodes of CD development. Thus, targeting HIGs might provide a novel effective therapeutic method for CD treatment. In this paper, we utilized the Enrichr platform to pinpoint seven potential drugs targeting HIGs: FENRETINIDE, cyclophosphamide, diuron, CROTONALDEHYDE, Demecolcine, DMBA, and Tetradioxin. Among these options, FENRETINIDE stands out for its ability to impact diverse biological pathways, encompassing insulin resistance, glucose tolerance, autophagy, and cell growth, thereby exhibiting a broad spectrum of pharmacological effects on conditions such as diabetes, cancer, and neurological diseases, all while demonstrating limited toxicity [29]. Cyclophosphamide is applied to enhance the expectancy and quality of life of cancer patients. However, it is considered as a dose-limiting drug because of the accompanied neurotoxicity [30]. Diuron treatment shows promise in ameliorating mammary tumour incidence or multiplicity [31]. CROTONALDEHYDE, generated through lipid peroxidation, possesses the capacity to modulate inflammatory processes by triggering epigenetic modifications via DNA adduct formation [32]. Demecolcine, a classic inhibitor of spindle fibre formation during M phase, finds widespread application as a mitotic inhibitor and apoptosis inducer [33]. DMBA treatment can result in differential expression of immune-related genes in mammary gland tissues from Wistar-Kyoto and Wistar-Furth rats [34]. While Tetradioxin hasn't been directly employed for therapeutic purposes, recent studies have unveiled its considerable potential in regulating immune systems among HIV-infected individuals as well as those afflicted by COVID-19 [35, 36]. Given that these drugs find application in processes linked to inflammation, immune response, or tumorigenesis, and that the utilization of specific drugs correlates closely with the expression of immune-related genes, we subsequently employed the molecular docking method (MDM) to delve into the binding affinity between the aforementioned drugs and their associated HIGs. Finally, our result revealed that Tetradioxin could theoretically bind to all the HIGs with the highest binding affinity, which suggested that Tetradioxin might be a promising drug for anti-CD treatment.

Certainly, there are some limitations in the present study. Firstly, we constructed a diagnostic prediction model based on only 132 samples from the GEO database. Thus, a larger cohort of patients is needed to confirm. Secondly, the GEO database provides limited clinical information and patient genetic data. Finally, to further reveal the potential regulatory role of immune-related genes in CD, functional experiments will be required in the future.

## Conclusions

In summary, we used three machine learning algorithms to identify HIGs for CD and validated the diagnostic effect of these HIGs in two independent datasets. We constructed an IG score based on HIGs which could assess the risk of CD. Also, we constructed a novel ANN model for CD diagnosis based on HIGs. In addition, we investigated the regulatory effect of HIGs in the pathogenesis of CD and identified potential drugs targeting HIGs using the Enrichr platform and MDM. The present findings may help comprehend CD's pathogenesis and provide a new perspective for CD's diagnostic and treatment strategy.

## Methods

### Data acquisition and preprocessing

The workflow chart of this study is shown in Fig. 1. We queried CD-related datasets from the GEO database, and the datasets needs to fulfill the following three criteria to be included in the present study. First, in order to perform gene expression analysis, the dataset should contain unbiased gene expression data with intact annotation. Second, in order to conduct the clinic-related analyses, CD patients included in the dataset should have complete clinical information. Third, to ensure the reliability of the bioinformatic analyses, CD patients included in the training cohort and validation cohort should be different and independent. Considering the above criteria, we screened and downloaded two independent CD datasets, GSE11501 and GSE164883, from the GEO database (https://www.ncbi.nlm.nih.gov/geo/). The GSE11501 dataset contains 110 CD patients and 22 healthy controls from The United Kingdom of Great Britain and Northern Ireland. The probes were transformed into the corresponding gene symbols using the GPL6104 platform annotation information. The GSE164883 dataset contains 26 CD patients and 22 healthy controls from Germany. The probes were transformed into the corresponding gene symbols using the GPL10558 platform annotation information. The GSE11501 dataset is used as the training set, and the GSE164883 dataset is used as the validation set. Then, based on the ImmPort database (https://www.immport.org/resources), we downloaded a list of immunologically relevant genes, which has 2483 immune-related genes (Additional file 1: Table S7). We downloaded the 28 immune gene sets from the TISIDB database (http://cis.hku.hk/TISIDB/) (Additional file 1: Table S8).

### Differential analysis of gene expression

We compared the expression profiles of CD patients and healthy controls to identify the differentially expressed genes (DEGs) of two clusters using the R package "limma", with a *p* value < 0.05 as the criterion. The *p* values were calculated using the Wilcoxon rank sum test.

### Functional enrichment analysis

To clarify which biological processes and functions the 58 differentially expressed immune-related genes are enriched in, to better comprehend the pathogenesis of CD, and we performed Gene Ontology (GO) and Kyoto Encyclopedia of Genes and Genomes (KEGG) analysis of the 58 differentially expressed immune-related genes using the “clusterProfiler” package in R software [37].

### Selection of hub immune-related genes (HIGs)

We used 3 machine algorithms to identify HIGs, namely: randomForest, LASSO and SVM-RFE, as these machine learning approaches have been widely employed to analyse biological data and accurately identify hub genes in gene expression profiles [38]. Firstly, we used the RF algorithm of “randomForest” package, the LASSO algorithm of “glmnet” package and the SVM-RFE algorithm of “e1071” package for screening 58 differentially expressed immune-related genes to identify potential candidate genes [39–41]. Then, we used venn diagrams to intersect the candidate genes screened by the above 3 algorithms, and finally found 3 intersecting HIGs.

### ssGSEA and consensus clustering analysis

We utilized the R packages "GSVA" and "GSEABase" to conduct single-sample gene set enrichment analysis (ssGSEA), and using the ssGSEA algorithm to evaluate the immunological characteristics among CD patients, respectively. We first obtained 28 immune gene sets from the TISIDB database (http://cis.hku.hk/TISIDB/), and then performed ssGSEA based on these 28 immune gene sets, and the ssGSEA score of 28 immune gene sets in each CD patients were calculated. Based on the ssGSEAscore of 28 immune gene sets, we used “ConsensusClusterPlus” package to identify the immune subtypes of CD patients. Using the pam algorithm with euclidean distance, the samples were iterated 1000 times, with the k value increased from 2 to 9.

### Generation of immune genes score

In order to quantify the immune-related gene expression pattern of celiac disease patients, we constructed a set of scoring systems—the immune genes score, which we termed as IG score. The procedures for establishment of IG score were as follows:

In order to construct IG score, principal component analysis (PCA) was performed based on the expression levels of HIGs and principal component 1 and principal component 2 were used as feature scores. The formula for calculating IG score is shown as follows:

$${\text{IG score}} = \sum \left( {{\text{PC}}1_{i} + {\text{PC}}2_{i} } \right)$$

In the formula, “*i*” represents HIGs. We grouped samples with IG score > 0 as high-IG score group and samples with IG score ≤ 0 as low-IG score group [42, 43].

### Construction and validation of the ANN model

We constructed the ANN model using HIGs, which was built using the R package “neuralnet” and consists of 3 parts:

- Input layer, which includes the gene expression of 3 HIGs;
- The first hidden layer, which includes the gene expressions of the 3 HIGs and the weights of the 3 HIGs; and the second hidden layer, which includes the weights of all neurons in hidden layer 1.
- Output layer, which indicates whether the sample belongs to healthy control or celiac disease.

To speed up the convergence and improve the accuracy of the ANN, and the first hidden layer was set to 6 neurons and the second hidden layer was set to 2, and ROC is used to evaluate the prediction performance of the ANN.

### Gene set enrichment analysis

To further identify which biological functions and signalling pathways are associated with HIGs, gene set enrichment analysis (GSEA) was performed on different subgroups of CD patients according to their median expression level of HIGs, with *P *< 0.05 considered statistically significant.

### Selection and docking of drugs targeting HIGs

To screen the drugs targeting HIGs, we used the Enrichr platform (https://maayanlab.cloud/Enrichr/) for online analysis and screening. First, we input the gene symbol of HIGs in the primary webpage of Enrichr platform, and then screened the drugs targeting HIGs based on the DSigDB database in the “Diseases/Drugs” module, and with *P *< 0.05 being statistically significant. Subsequently, we used molecular docking method (MDM) to investigate the interaction and binding affinity of the screened drug molecules to their HIGs in order to screen for the most potential drugs. Specifically, the protein (*CCL25*, *MR1* and *TNFSF13B*) that was used in this study was deposited in the NCBI-Protein databases under the accession number of O15444, Q95460 and Q9Y275. The validation and quality estimation of predicted *CCL25*, *MR1* and *TNFSF13B* model were evaluated by PROCHECK and QMEAN, respectively [44, 45]. AutoDock tools were used to prepare the ligand and protein files [46]. Protein–ligand docking was performed with AutoDock tools, and the resulting interactions between receptor and ligand were visualized with PyMOL (version 2.5) and LigPlus (version 2.2) [47].

### Statistical analysis

Statistical analysis and visualization were conducted using R software for this study. The analysis of variance (ANOVA) method was employed to statistically analyse multi-group data, while the wilcoxon rank sum test was used to compare two groups. The association between the ssGSEA score and IG score was assessed using spearman's correlation coefficient. Statistical significance was defined as *P *< 0.05 for all statistical analyses.

### Supplementary Information

**Additional file 1: Table S1.** 896 differentially expressed genes. **Table S2.** Results of three machine algorithms. **Table S3.** ssGSEA score of 28 immune gene sets in celiac disease patients. **Table S4.** IG score for GSE11501 training set based on HIGs. **Table S5.** IG score for GSE164883 validation set based on HIGs. **Table S6. **ANN diagnosis effect for the grouping of immune characteristics of celiac disease subtypes. **Table S7.** 2483 immune genes from the ImmPort database. **Table S8.** 28 immune gene sets from the TISIDB database.

**Additional file 2: Fig. S1.** GO and KEGG analysis of 58 differentially expressed immune-related genes. **A** GO enrichment results in differentially expressed immune-related genes. **B** KEGG enrichment results in differentially expressed immune-related genes. **Fig. S2.** Heatmap shows the overall landscape of CD patients' ssGSEA score of 28 immune gene sets. **Fig. S3.** Consensus matrix heatmap when K = 3–9. It is related to Fig. 3D. **Fig. S4.** The box plot shows the ssGSEA score of immune cells of the C1 and C2 groups. (ns, no significance, *P < 0.05, **P < 0.01, ***P < 0.001). **Fig. S5.** Validation of the IG score in the GSE164883 set. **A** The violin plot shows the IG score between the control and CD groups. **B** The ROC curve of the IG score in the GSE164883 validation set. **Fig. S6.** ROC analysis validated the diagnostic performance of HIGs. ROC curves of the indicated HIGs in the GSE11501 training set (**A**) and GSE164883 validation set (**B**). **Fig. S7.** Construction of artificial neural network (ANN) based on HIGs. **A** The construction of an artificial neural network (ANN) based on *MR1*, *TNFSF13B*, and *CCL25*. **B** The AUC of the training cohort with a value of 0.824. **C** The AUC of the test cohort with a value of 0.733. **Fig. S8.** 3D (left) and 2D (right) structure of complexes of HIGs and drugs. It is related to Fig. 7.

## Data Availability

Datasets related to this article are from public database (GSE11501 and GSE164883). All data generated or analysed during this study are included in this article/Additional files, further inquiries can be directed to the corresponding author.

## Abbreviations

CD: Celiac disease
HC: Healthy control
GEO: Gene expression omnibus
DEGs: Differentially expressed genes
GO: Gene ontology
KEGG: Kyoto Encyclopedia of Genes and Genomes
GFD: Gluten-free diet
HIGs: Hub immune-related genes
IG score: Immune genes score
RF: RandomForest
SVM-RFE: Support vector machine recursive feature elimination
LASSO: Last absolute shrinkage and selection operator
ANN: Artificial neural network
ssGSEA: Single-sample gene set enrichment analysis
GSEA: Gene set enrichment analysis
MDM: Molecular docking method
HLA: Human leukocyte antigen
PCA: Principal component analysis
*MR1*: Major histocompatibility complex class I-related gene
*CCL25*: C-C Motif chemokine ligand 25
*TNFSF13B*: TNF superfamily member 13b
ROC: Receiver operating characteristic
AUC: Area under the ROC curve
ANOVA: Analysis of variance
GI: Gastrointestinal
UMAP: Uniform manifold approximation and projection
t-SNE: T-distributed stochastic neighbor embedding
C1: Cluster 1
C2: Cluster 2
DCs: Dendritic cells
MAIT: Mucosal associated invariant T cells
TNF: Tumor necrosis factor
INF: Interferon
IL: Interleukin
LEU: Leucine
TRP: Tryptophan
LYS: Lysine
TYR: Tyrosine
ASN: Asparagine
PRO: Proline
PHE: Phenylalanine
ILE: Isoleucine
GLN: Glutamine
ALA: Alanine
