# Supplementary material for: Developing neural network diagnostic models and potential drugs based on novel identified immune-related biomarkers for celiac disease
Source: Hum Genomics. 2023 Aug 17;17:76. doi: 10.1186/s40246-023-00526-z (PMC10433645; doi:10.1186/s40246-023-00526-z)
Supplement: Supplementary file 2 — Additional file 2: Fig. S1. GO and KEGG analysis of 58 differentially expressed immune-related genes. A GO enrichment results in differentially expressed immune-related genes. B KEGG enrichment results in differentially expressed immune-related genes. Fig. S2. Heatmap shows the overall landscape of CD patients' ssGSEA score of 28 immune gene sets. Fig. S3. Consensus matrix heatmap when K = 3–9. It is related to Fig. 3D. Fig. S4. The box plot shows the ssGSEA score of immune cells of the C1 and C2 groups. (ns, no significance, *P < 0.05, **P < 0.01, ***P < 0.001). Fig. S5. Validation of the IG score in the GSE164883 set. A The violin plot shows the IG score between the control and CD groups. B The ROC curve of the IG score in the GSE164883 validation set. Fig. S6. ROC analysis validated the diagnostic performance of HIGs. ROC curves of the indicated HIGs in the GSE11501 training set (A) and GSE164883 validation set (B). Fig. S7. Construction of artificial neural network (ANN) based on HIGs. A The construction of an artificial neural network (ANN) based on MR1, TNFSF13B, and CCL25. B The AUC of the training cohort with a value of 0.824. C The AUC of the test cohort with a value of 0.733. Fig. S8. 3D (left) and 2D (right) structure of complexes of HIGs and drugs. It is related to Fig. 7. [file 40246_2023_526_MOESM2_ESM.docx]

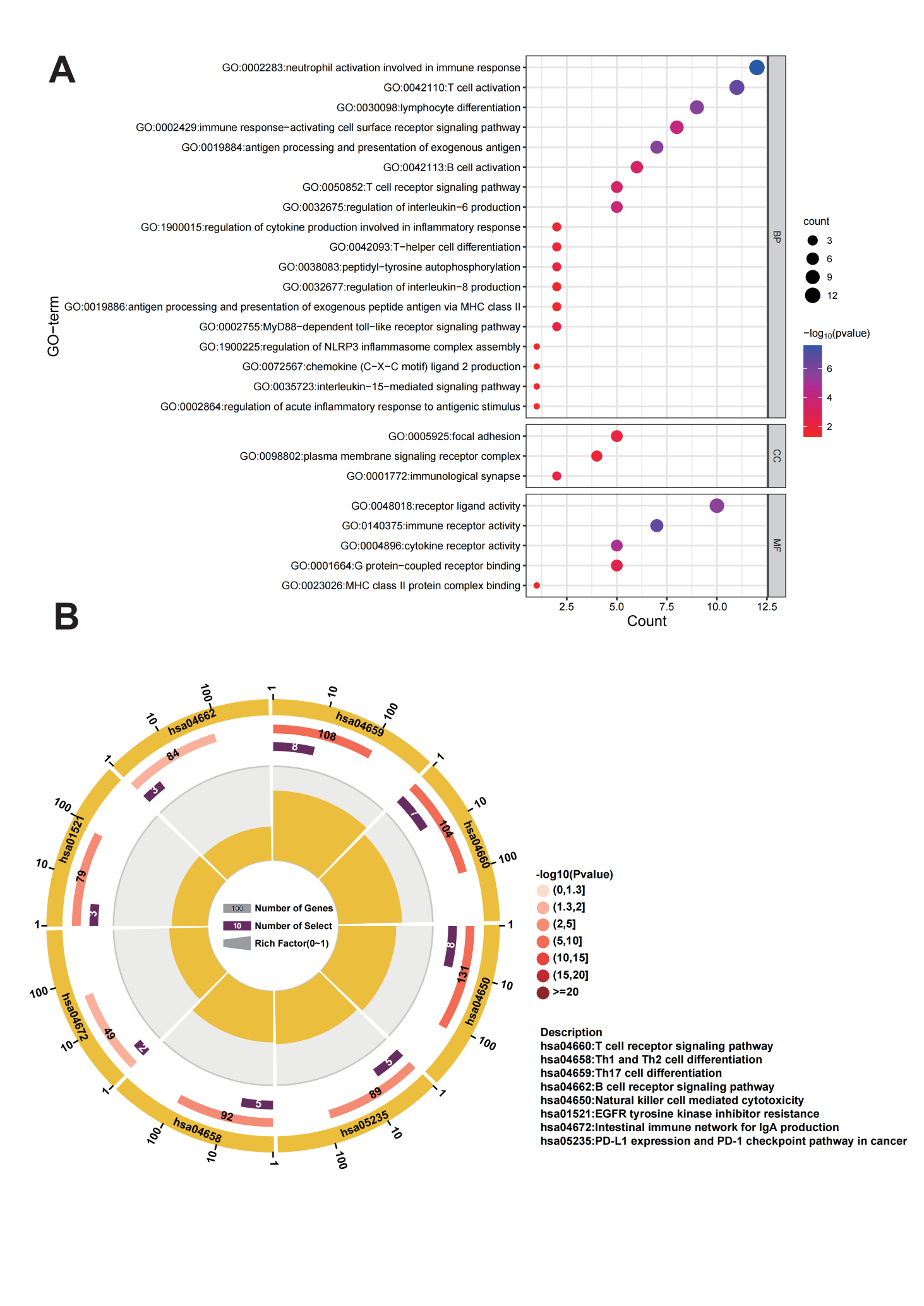


**Figure S1.** GO and KEGG analysis of 58 differentially expressed immune-related genes. **(A)** GO enrichment results in differentially expressed immune-related genes. **(B)** KEGG enrichment results in differentially expressed immune-related genes.

.
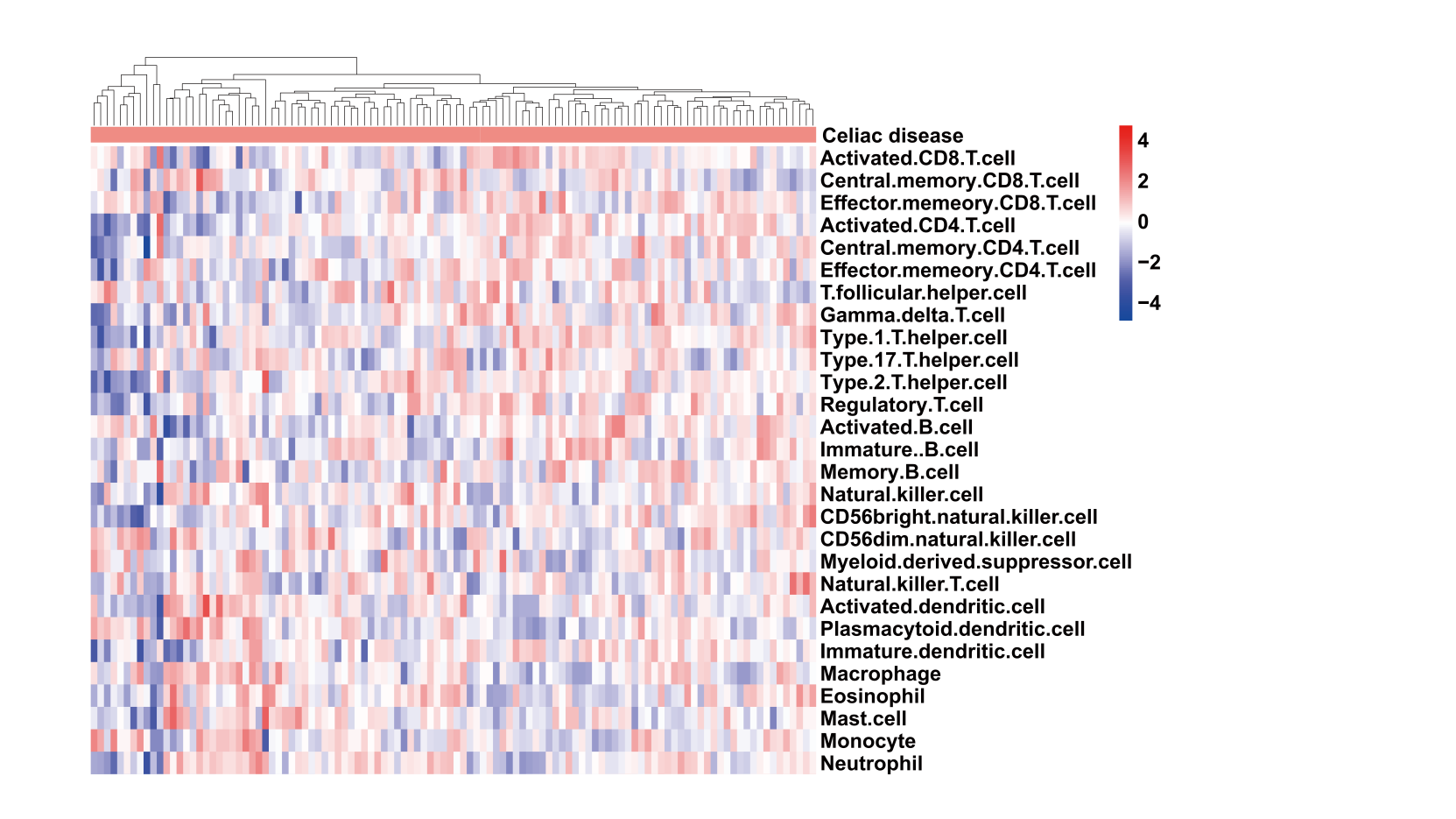


**Figure S2.** Heatmap shows the overall landscape of CD patients' ssGSEA score of 28 immune gene sets.


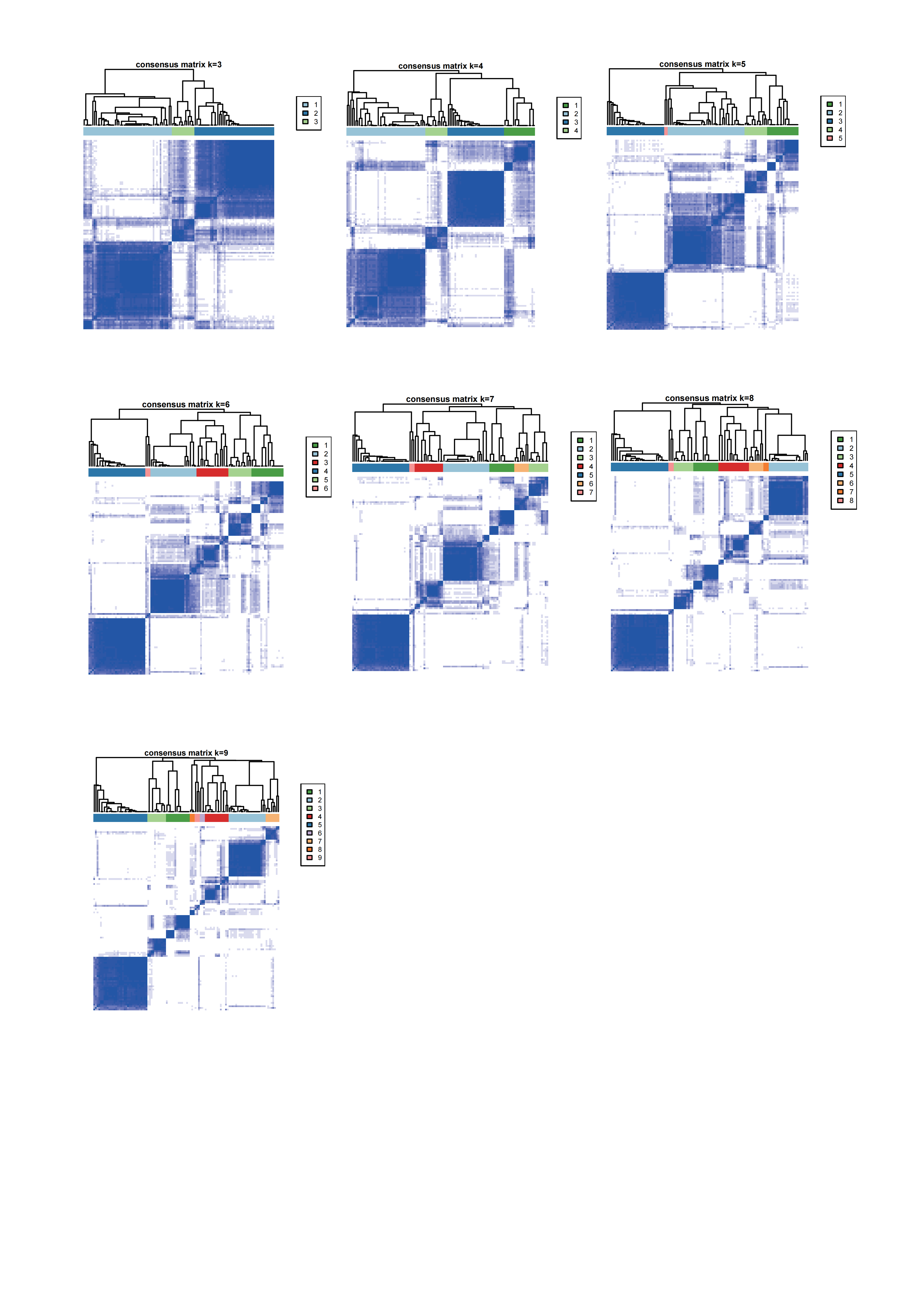


**Figure S3.** Consensus matrix heatmap when K = 3-9. It is related to figure 3D.


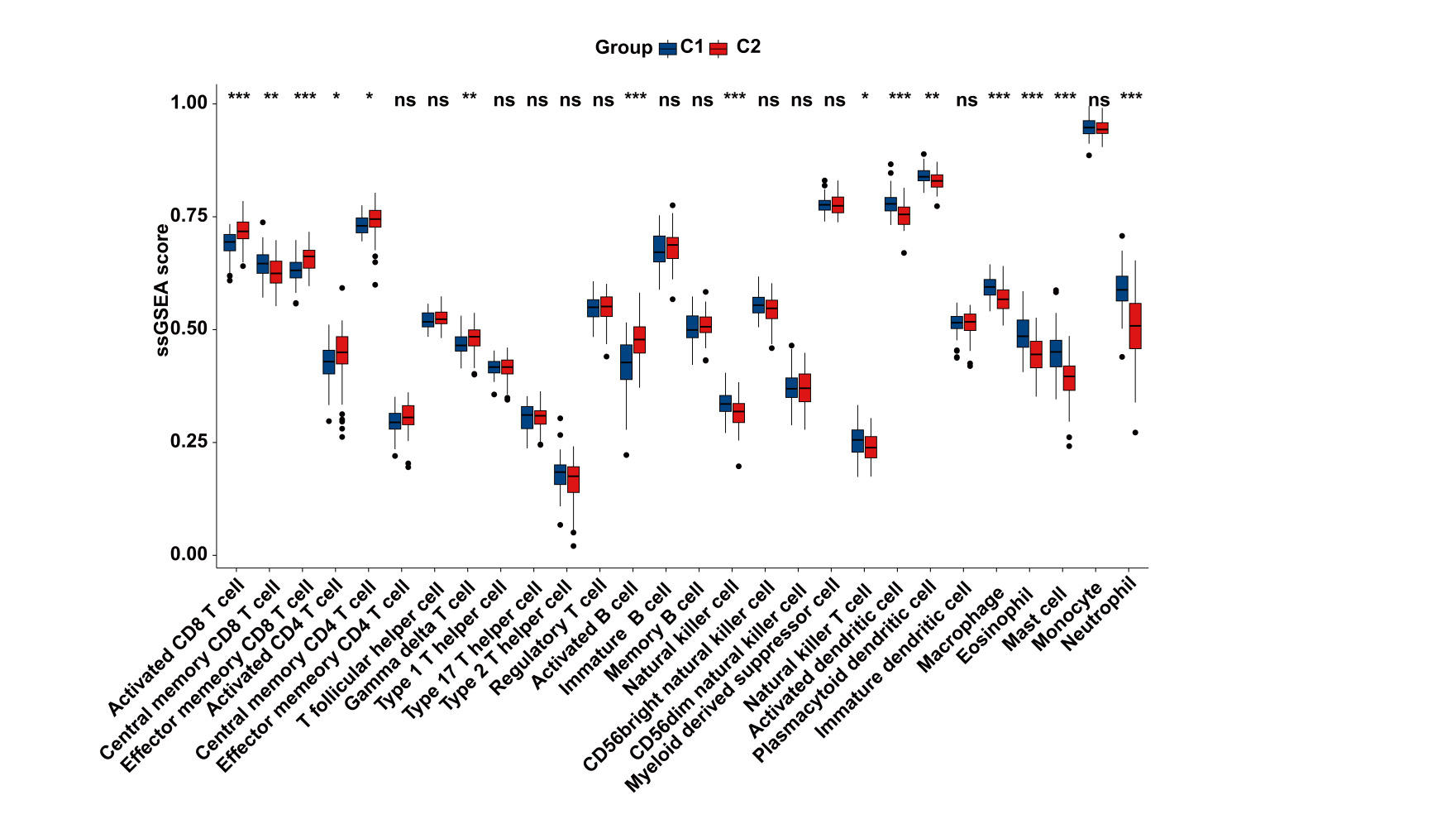


**Figure S4.** The box plot shows the ssGSEA score of immune cells of the C1 and C2 groups. (ns, no significance, *P < 0.05, **P < 0.01, ***P < 0.001).


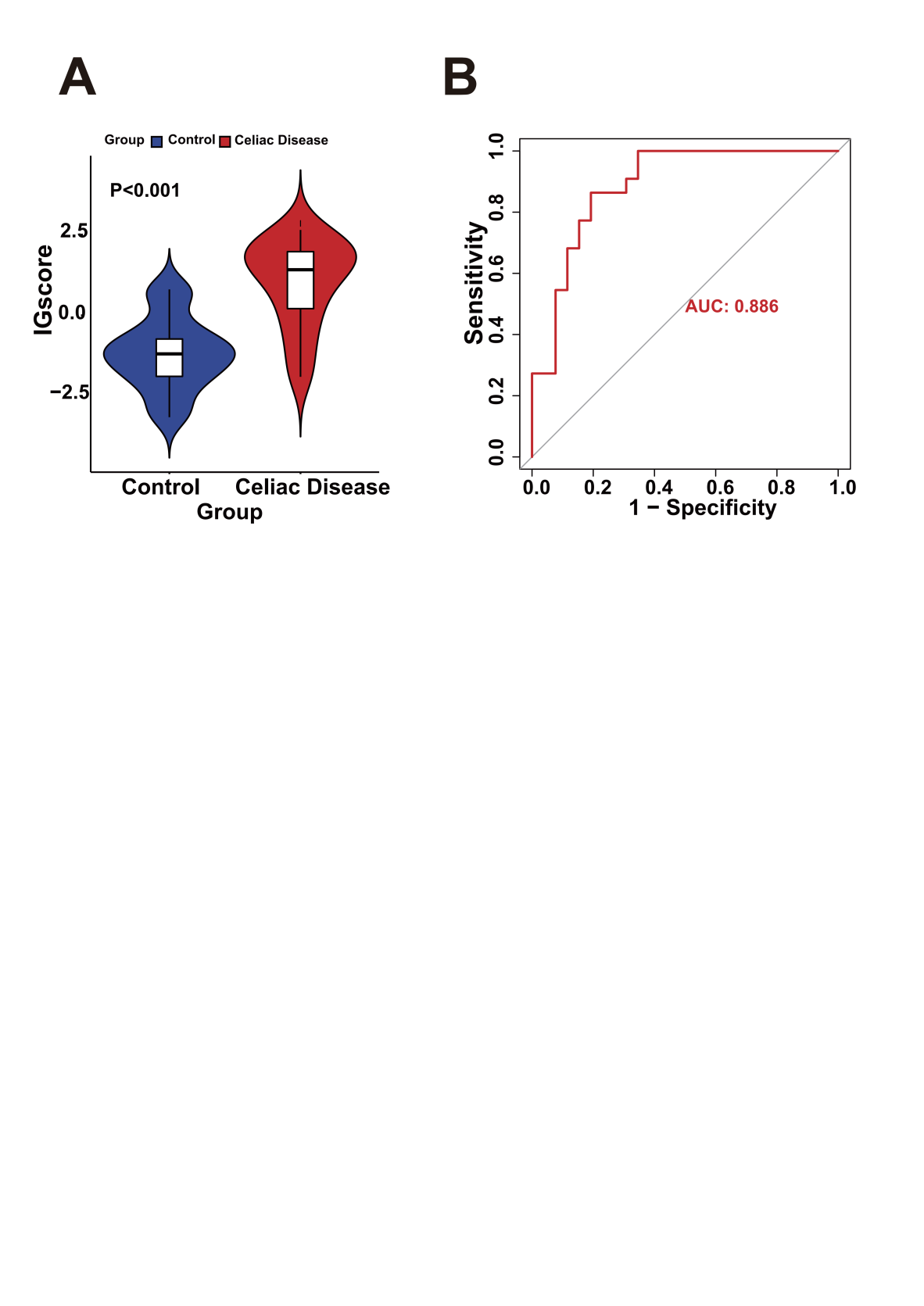


**Figure S5.** Validation of the IG score in the GSE164883 set. **(A)** The violin plot shows the IG score between the control and CD groups. **(B)** The ROC curve of the IG score in the GSE164883 validation set.


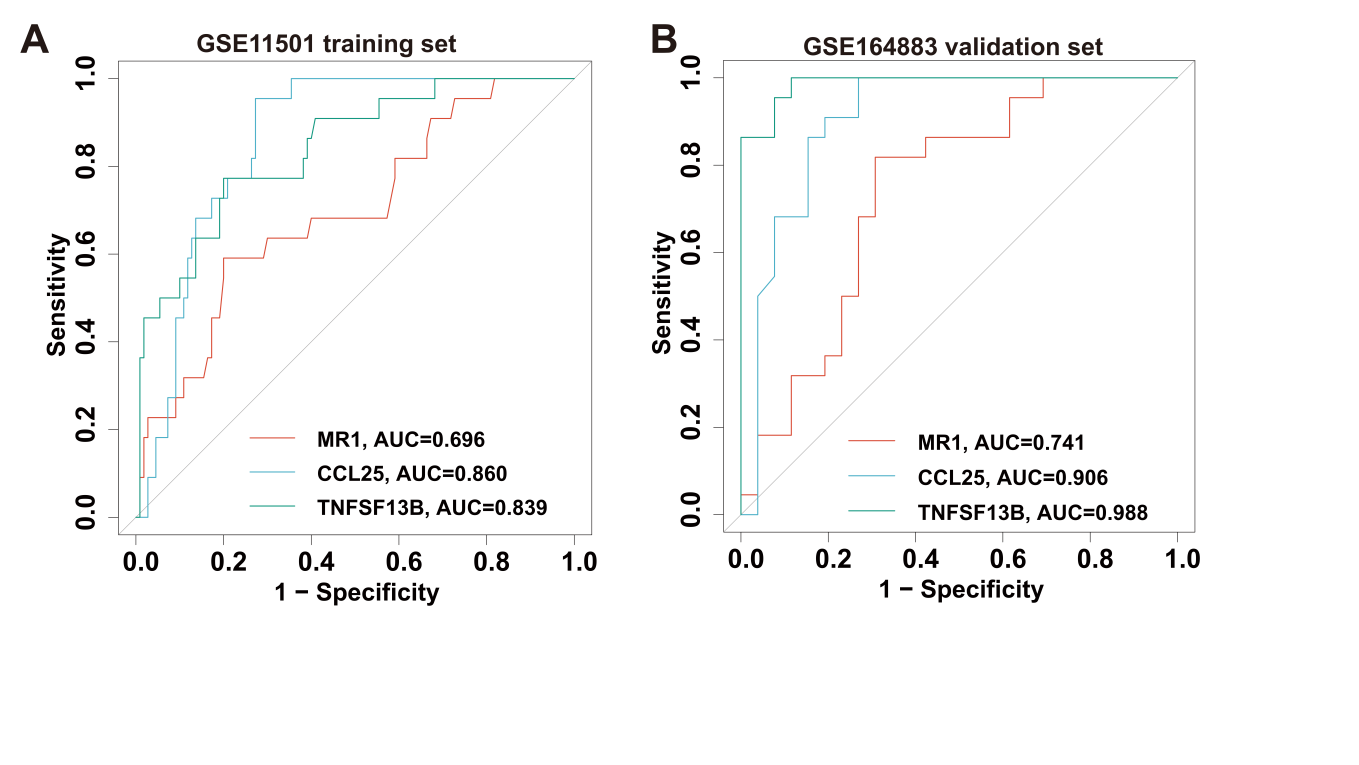


**Figure S6.** ROC analysis validated the diagnostic performance of HIGs. ROC curves of the indicated HIGs in the GSE11501 training set (A) and GSE164883 validation set (B).


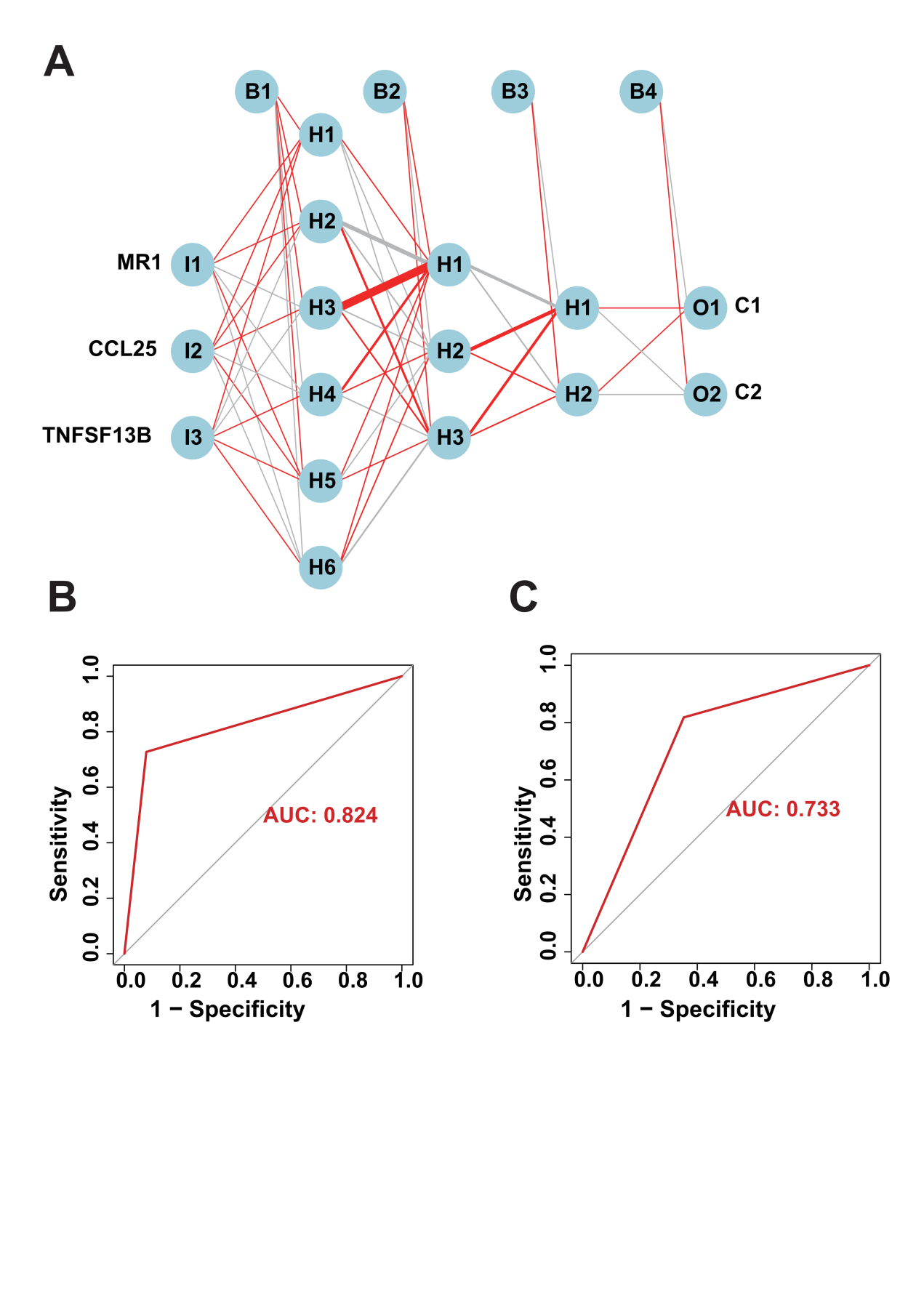


**Figure S7.** Construction of artificial neural network (ANN) based on HIGs. **(A)** The construction of an artificial neural network (ANN) based on *MR1*, *TNFSF13B*, and *CCL25*. **(B)** The AUC of the training cohort with a value of 0.824. **(C)** The AUC of the test cohort with a value of 0.733.


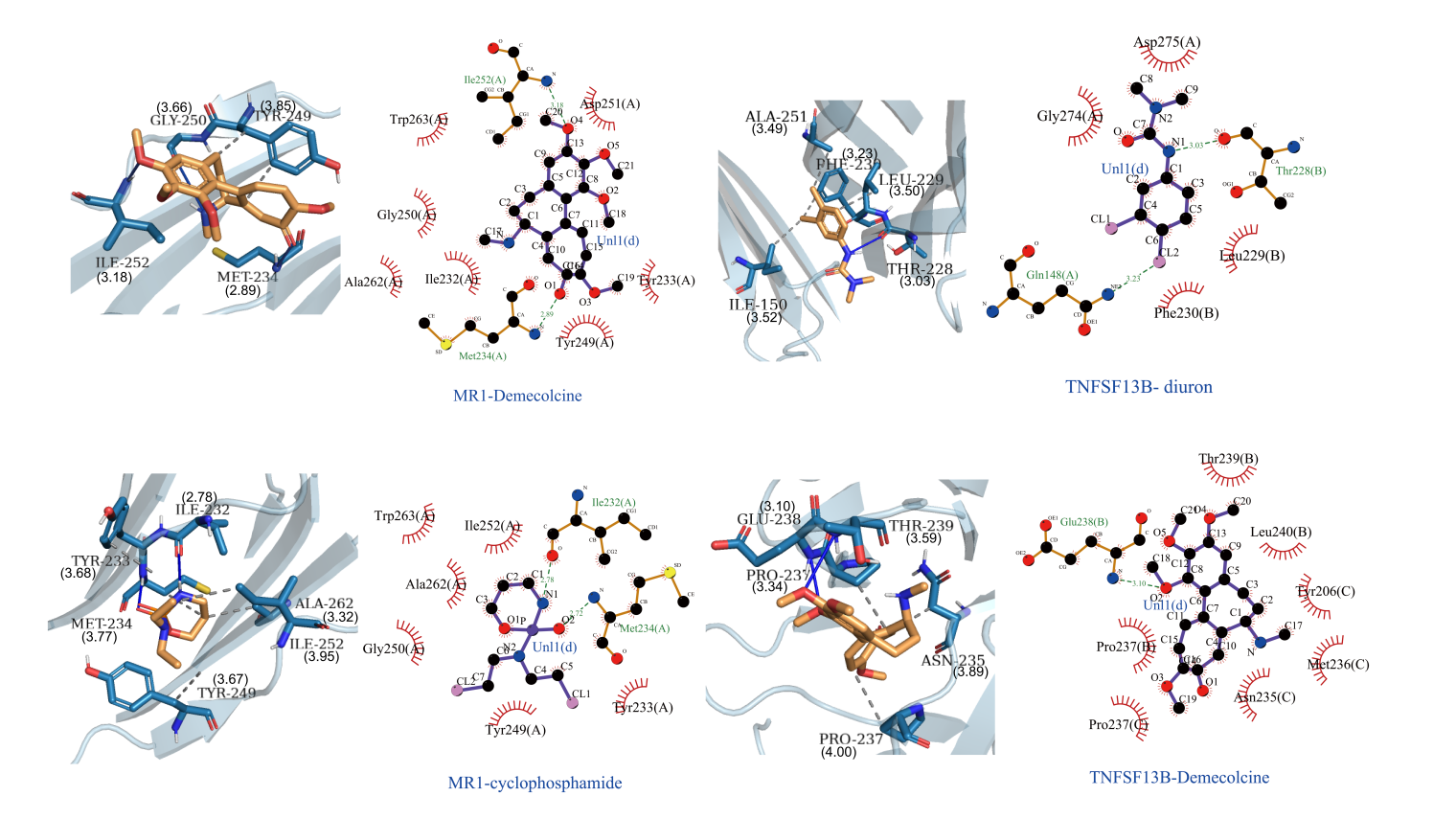


**Figure S8.** 3D (left) and 2D (right) structure of complexes of HIGs and drugs. It is related to Figure 7.
